# Reporting Quality of Systematic Reviews and Meta-Analyses of Otorhinolaryngologic Articles Based on the PRISMA Statement

**DOI:** 10.1371/journal.pone.0136540

**Published:** 2015-08-28

**Authors:** Jeroen P. M. Peters, Lotty Hooft, Wilko Grolman, Inge Stegeman

**Affiliations:** 1 Department of Otorhinolaryngology and Head & Neck Surgery, University Medical Center Utrecht, Utrecht, The Netherlands; 2 Brain Center Rudolf Magnus, University Medical Center Utrecht, Utrecht, The Netherlands; 3 Dutch Cochrane Centre, Julius Center for Health Sciences and Primary Care, University Medical Center Utrecht, Utrecht, The Netherlands; Liverpool School of Tropical Medicine, UNITED KINGDOM

## Abstract

**Background:**

Systematic reviews (SRs) and meta-analyses (MAs) provide the highest possible level of evidence. However, poor conduct or reporting of SRs and MAs may reduce their utility. The PRISMA Statement (*Preferred Reporting Items for Systematic reviews and Meta-Analyses*) was developed to help authors report their SRs and MAs adequately.

**Objectives:**

Our objectives were to (1) evaluate the quality of reporting of SRs and MAs and their abstracts in otorhinolaryngologic literature using the PRISMA and PRISMA for Abstracts checklists, respectively, (2) compare the quality of reporting of SRs and MAs published in Ear Nose Throat (ENT) journals to the quality of SRs and MAs published in the ‘gold standard’ Cochrane Database of Systematic Reviews (CDSR), and (3) formulate recommendations to improve reporting of SRs and MAs in ENT journals.

**Methods:**

On September 3, 2014, we searched the Pubmed database using a combination of filters to retrieve SRs and MAs on otorhinolaryngologic topics published in 2012 and 2013 in the top 5 ENT journals (ISI Web of Knowledge 2013) or CDSR and relevant articles were selected. We assessed how many, and which, PRISMA (for Abstracts) items were reported adequately per journal type.

**Results:**

We identified large differences in the reporting of individual items between the two journal types with room for improvement. In general, SRs and MAs published in ENT journals (n = 31) reported a median of 54.4% of the PRISMA items adequately, whereas the 49 articles published in the CDSR reported a median of 100.0 adequately (difference statistically significant, *p* < 0.001). For abstracts, medians of 41.7% for ENT journals and 75.0% for the CDSR were found (*p* < 0.001).

**Conclusion:**

The reporting of SRs and MAs in ENT journals leaves room for improvement and would benefit if the PRISMA Statement were endorsed by these journals.

## Introduction

Systematic reviews (SRs) and meta-analyses (MAs) have the highest possible level of evidence in medical literature [1]. Both SRs and MAs combine the results of a number of trials. Poorly conducted primary trials, even randomised controlled trials (RCTs), could lead to the introduction of bias (systematic inclination inhibiting impartial judgment). Bias may reduce the utility of SRs and MAs. Clear presentation of what was planned, done and found in SRs and MAs is essential to value its findings, because clinicians use the results directly in clinical care.

In 1999 the QUORUM Statement (*QUality Of Reporting Of Meta-analyses*) was developed to help authors report meta-analyses adequately [2]. In 2009, the QUORUM Statement was replaced by the PRISMA Statement (*Preferred Reporting Items for Systematic reviews and Meta-Analyses*) to also apply to SRs [3, 4]. Adherence to the QUORUM [5] or PRISMA [6–10] Statements improved the quality of reporting of published SRs and MAs.

To our knowledge, the quality of reporting of SRs and MAs has not been assessed in otorhinolaryngologic literature yet. In current medicine, where evidence-based medicine is taking a prominent place, adequate reporting of the findings of SRs and MAs is important, also for clinical practice and patient care. Therefore, our primary aim was to assess the quality of reporting of (abstracts of) SRs and MAs in otorhinolaryngologic literature. Our second aim was to compare the quality of reporting of SRs and MAs published in Ear Nose Throat (ENT) journals to otorhinolaryngologic SRs and MAs published in the Cochrane Database of Systematic Reviews (CDSR). Finally, we formulated recommendations to improve the reporting of SRs and MAs in otorhinolaryngology.

## Methods

### Journals

We included SRs from the top 5 ENT journals, based on ISI Web of Knowledge 2013 impact factors (www.webofknowledgde.com). The top 5 ENT journals are *Head & Neck* (Head Neck), *Hearing Research* (Hear Res), *Ear & Hearing* (Ear Hear), *Rhinology* and *Journal of the Association for Research in Otolaryngology* (JARO) (Table 1). None of these top 5 ENT journals endorse the PRISMA Statement in their instructions to authors (evaluated on November 6^th^, 2014). To compare the quality of reporting of SRs and MAs published in ENT journals to the ‘gold standard’ of systematic reviews of the literature, SRs and MAs published in the CDSR were extracted. The CDSR does endorse the PRISMA Statement.

**Table 1:** Impact Factors (2013) top 5 Ear Nose Throat (ENT) journals and Cochrane Database of Systematic Reviews.

Journal	Impact Factor*
ENT journals (journal abbreviation)	
1. Head & Neck (Head Neck)	3.006
2. Hearing Research (Hear Res)	2.848
3. Ear & Hearing (Ear Hear)	2.833
4. Rhinology	2.779
5. Journal of the Association for Research in Otolaryngology (JARO)	2.547
Cochrane Database of Systematic Reviews	5.939

* Source: ISI Web of Knowledge 2013, Journal Citations Reports via www.webofknowledge.com, accessed on September 3^rd^, 2014.

### Search

We performed a Pubmed search using five filters. First, an adapted version of the Cochrane ENT search filter was used to retrieve otorhinolaryngologic articles (S1 File) [11]. Second, to retrieve only SRs and MAs, the Pubmed filter for SRs and MAs was used [12]. Third, a date restriction was applied to retrieve only articles indexed in 2012 and 2013. Fourth, a filter was used to search only in the top 5 ENT journals and a filter was used to search only in the CDSR. Fifth, editorials, letters, news and comments as publication type were excluded (for complete search: see S1 File). Finally, a combination was made with a search syntax for the ENT journals and the CDSR using Boolean operator AND.

### Study selection

Two authors (JPMP and IS) independently assessed titles, abstracts and full texts of the retrieved articles to check if the study was indeed a SR or MAs and if it was conducted in the otorhinolaryngologic field. To be considered as a study in the otorhinolaryngologic field, studies must assess patient populations generally treated by otorhinolaryngologists or investigate a procedure generally performed by otorhinolaryngologists, including head and neck surgery (S1 File).

Throughout this paper, we adopt the definition of SRs and MAs of the PRISMA Statement [3] and Cochrane Collaboration (http://handbook.cochrane.org): “a systematic review is a review of a clearly formulated question that uses systematic and explicit methods to identify, select, and critically appraise relevant research, and to collect and analyze data from the studies that are included in the review. Statistical methods (meta-analysis) may or may not be used to analyze and summarize the results of the included studies. Meta-analysis refers to the use of statistical techniques in a systematic review to integrate the results of included studies”.

Discrepancies between the two reviewers were discussed until consensus was reached. When no consensus could be reached, an independent otorhinolaryngologist was consulted.

### PRISMA Statement adherence

The PRISMA 2009 checklist was used to score the quality of reporting [3]. The included articles were read and assessed independently by two authors (JPMP and IS). We evaluated the number of items of the PRISMA checklist that were adequately reported. The total number of items on the PRISMA checklist is 27, however, item 2 (Abstract) was scored separately (see *PRISMA for Abstracts adherence*). Some items of the PRISMA checklist (item 14, 16, 21 and 23) are specific for meta-analysis only. They were not scored as missing or inadequately reported when not applicable. A more detailed explanation on how items were assessed can be found in S2 File. Differences in opinion were discussed until consensus was reached.

### PRISMA for Abstracts adherence

The abstracts of the included SRs and MAs were assessed using the PRISMA for Abstracts checklist [13]. The PRISMA for Abstracts checklist is a detailed checklist of what items should be reported in abstracts of SRs and MAs. The total number of items on the PRISMA for Abstracts checklist is 12. A more detailed explanation on how all 12 items were assessed can be found in S3 File.

### Data analysis

We calculated descriptive statistics per item of the PRISMA checklist and the PRISMA for Abstracts checklist. Using the Chi2 test, we evaluated if there was a statistically significant difference between the two journal types in the reporting per item.

Furthermore, we divided the number of adequately reported PRISMA items by a possible total of 26 items (Item 2, Abstract, was scored separately) if the assessed paper was a MA and divided by a possible total of 22 items if the assessed paper was a SR, resulting in a percentage. The higher the percentage, the more adequately the SR or MA was reported. For the assessment of the abstracts, the number of adequately reported PRISMA for Abstracts items was divided by a possible total of 12. Subsequently, we calculated the median and mean percentages (and 95% confidence intervals (CI)) per journal type to be able to compare the reporting of specific PRISMA (for Abstracts) items between the SRs and MAs published in ENT journals and in the CDSR. The 2-tailed Mann Whitney U test for 2 independent samples was used to compare PRISMA (for Abstracts) scores for SRs and MAs published in ENT journals and in CDSR.

Statistical tests were performed using SPSS v20 statistics package. A *p*-value of < 0.05 was considered statistically significant.

## Results

### Search

**Fig 1:**
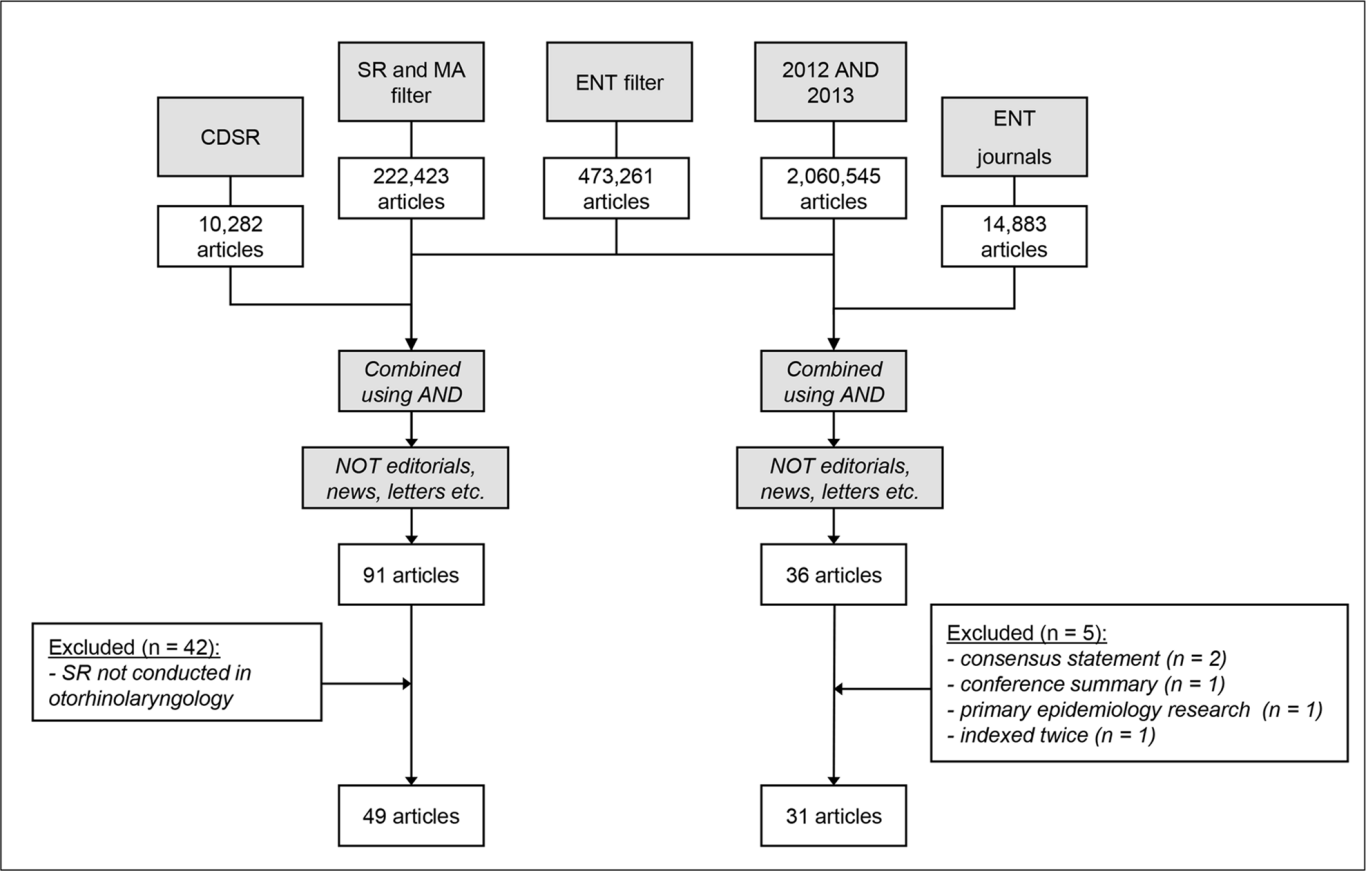
Flowchart of search (date of search: 3 September 2014). SR = Systematic Review, MA = meta-analysis, ENT = Ear, Nose, Throat, CDSR = Cochrane Database of Systematic Reviews.

The search process is shown in Fig 1. The combined search syntaxes yielded 36 articles from ENT journals and 91 articles from the CDSR (S1 File).

### Study selection

All 36 articles published in ENT journals qualified as studies that were conducted in the otorhinolaryngologic research field. Not all retrieved articles were true SRs or MAs (i.e. the Pubmed filter is not 100% specific for SRs and MAs): 2 papers were consensus statements, 1 paper was a summary of a conference, 1 paper was an epidemiologic paper including SRs and 1 paper was indexed twice; the latest published version of this article was included. The remaining 31 articles (Head Neck n = 24, Hear Res n = 1, Ear Hear n = 2, Rhinology n = 4, JARO n = 0) were further analysed, 26 were SRs and 5 were MAs.

All 91 articles published in CDSR were SRs or MAs. Of these, 42 did not qualify as studies conducted in the otorhinolaryngologic research field. The remaining 49 articles were included for data analysis. Of these, 10 were pure SRs and 39 also conducted a meta-analysis. These 49 SRs were conducted in large part by the Cochrane Ear, Nose and Throat Disorders group (n = 25), but also by the Acute Respiratory Infections group (n = 18), the Anaesthesia group (n = 2), the Childhood Cancer group, the Occupational Safety and Health group, the Developmental, Psychosocial and Learning Problems group, and the Cystic Fibrosis and Genetic Disorders group (each n = 1).

### PRISMA

**Fig 2:**
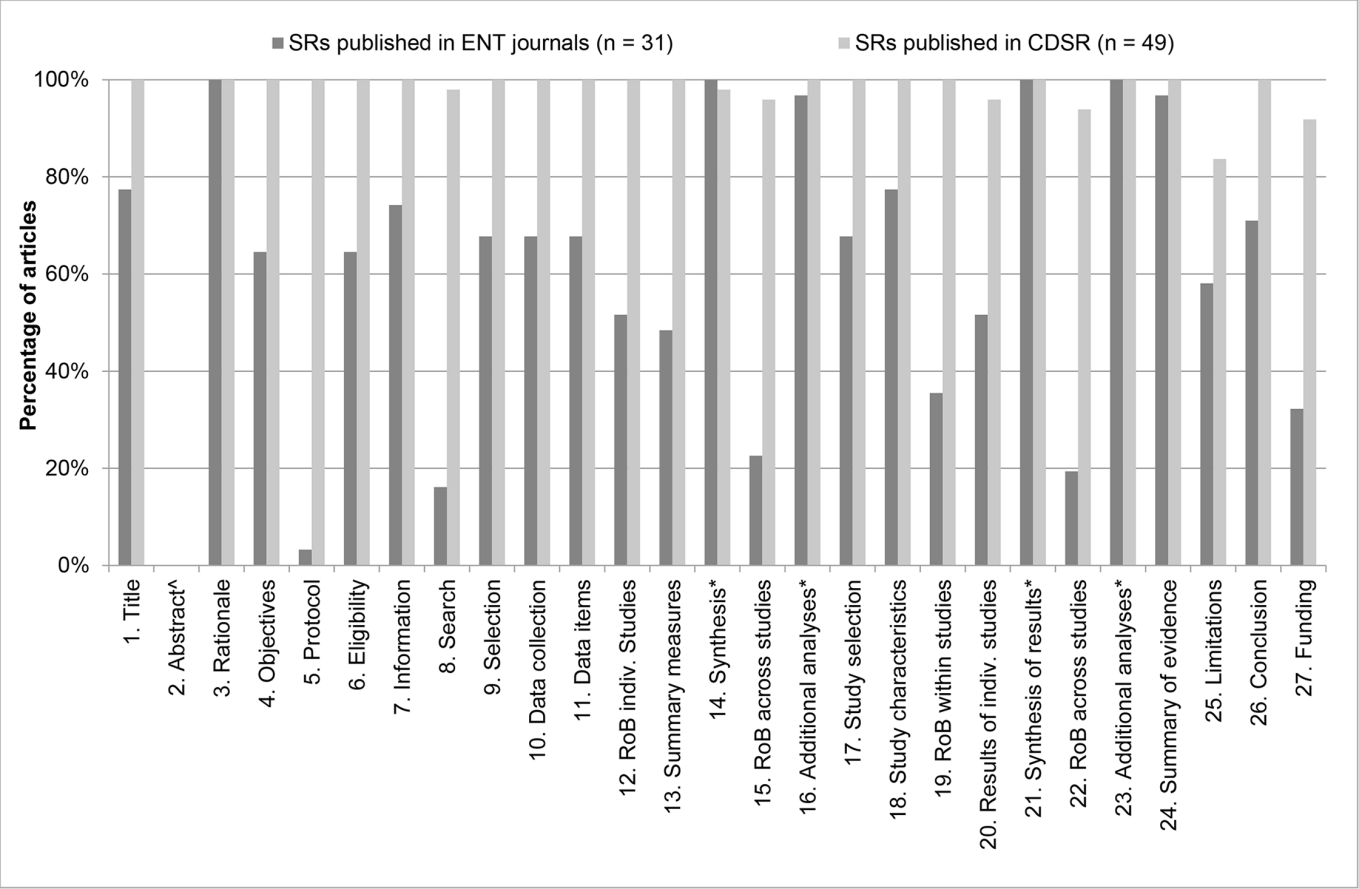
Number of adequately reported PRISMA items per journal type. The percentage of articles that adequately reported PRISMA items is plotted per journal type (ENT journals [dark grey bars]-vs- CDSR [light grey bars]). For exact percentages, see Table 2. ^ Item 2 is scored separately, see Fig 3. Items 14, 16, 21 and 23 are optional items. For details on scoring, see S2 File.

The exact percentages of adequately reported PRISMA items for SRs and MAs published in ENT journals and CDSR are presented in Table 2, as well as the significance of the difference between the two journal types. A graphic illustration is provided in Fig 2.

**Table 2:** Data table of Fig 2, number of adequately reported PRISMA items per journal type.

**Item**	**1**	**2** ^	**3**	**4**	**5**	**6**	**7**	**8**	**9**	**10**	**11**	**12**	**13**	
SRs published in ENT journals (n = 31)	77.4%		100.0%	64.5%	3.2%	64.5%	74.2%	16.1%	67.7%	67.7%	67.7%	51.6%	48.4%	
SRs published in CDSR (n = 49)	100.0%		100.0%	100.0%	100.0%	100.0%	100.0%	98.0%	100.0%	100.0%	100.0%	100.0%	100.0%	
p-value^$^	<0.001		NA	<0.001	<0.001	<0.001	<0.001	<0.001	<0.001	<0.001	<0.001	<0.001	<0.001	
**Item**	**14** *	**15**	**16** *	**17**	**18**	**19**	**20**	**21** *	**22**	**23** *	**24**	**25**	**26**	**27**
SRs published in ENT journals (n = 31)	100.0%	22.6%	96.8%	67.7%	77.4%	35.5%	51.6%	100.0%	19.4%	100.0%	96.8%	58.1%	71.0%	32.3%
SRs published in CDSR (n = 49)	98.0%	95.9%	100.0%	100.0%	100.0%	100.0%	95.9%	100.0%	93.9%	100.0%	100.0%	83.7%	100.0%	91.8%
p-value^$^	*0*.*423*	<0.001	0.206	<0.001	<0.001	<0.001	<0.001	NA	<0.001	NA	*0*.*206*	0.011	<0.001	<0.001

The percentage of articles that adequately reported PRISMA items per journal type, ENT journals-vs- CDSR.

^ Item 2 is scored separately, see Table 3.

^$^ Chi2 test. P-values in italic typeface highlight a difference that was *not* statistically significantly different between the two journal types. NA = not applicable.

* Optional items, e.g. “if done”. If possible in the study and adequately reported, the item was scored as ‘adequately reported’. If possible, but not reported, the item was scored as ‘inadequately reported’. If not possible, the item was not scored as ‘inadequately reported’.

SRs and MAs published in ENT journals reported several individual items inadequately more frequently than SRs and MAs published in the CDSR. First, only 3% of SRs published in ENT journals refer to a published review protocol of their studies (item 5). Second, less than 20% of SRs or MAs report their full search syntax (item 8), either in the main text or in online supplementary material. Third, more than three quarters of the studies did not report the assessment of risk of bias across studies (items 15 and 22) inadequately, possibly reflecting that the risk of bias across studies was not considered in their reviews. Moreover, the articles fail to report an assessment of the risk of bias within the included studies adequately (item 19). Finally, more than two thirds of the articles do not report their source of funding, thereby omitting possible conflicts of interest (item 27). Unlike the articles published in ENT journals, SRs from the CDSR reported a vast majority of PRISMA item adequately.

In sum, the 31 articles published in the top 5 ENT journals reported a median of 54.4% (mean 62.2%, 95% CI: 54.4%-71.7%) of the PRISMA items adequately, whereas the 49 articles published in the CDSR reported a median of 100.0% (mean 98.2%, 97.3%-99.1%) adequately. The two journal types reported PRISMA items statistically significantly different (*p* < 0.001).

### PRISMA for Abstracts

**Fig 3:**
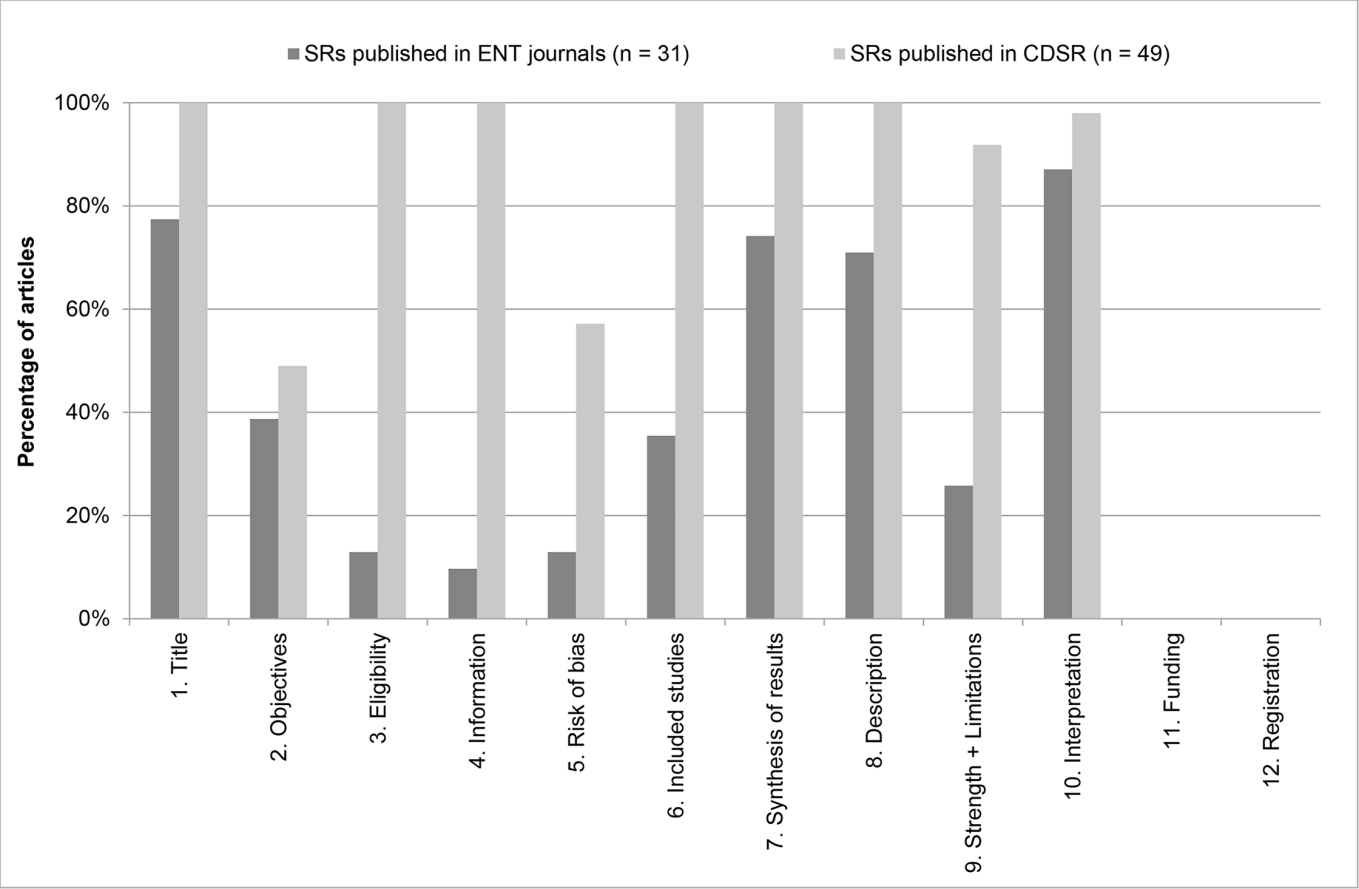
Number of adequately reported PRISMA for Abstract items per journal type. The percentage of articles that adequately reported PRISMA for Abstract items is plotted per journal type (ENT journals [dark grey bars]-vs- CDSR [light grey bars]). For exact percentages, see Table 3. For details on scoring, see S3 File.

The exact percentages of adequately reported PRISMA for Abstracts items for SRs and MAs published in ENT journals and CDSR are reported in Table 3 (together with statistical significance), with a graphic illustration in Fig 3.

**Table 3:** Data table of Fig 3, number of adequately reported PRISMA for Abstract items per journal type.

Item	1	2	3	4	5	6	7	8	9	10	11	12
SRs published in ENT journals (n = 31)	77,4%	38,7%	12,9%	9,7%	12,9%	35,5%	74,2%	71,0%	25,8%	87,1%	0,0%	0,0%
SRs published in CDSR (n = 49)	100,0%	49,0%	100,0%	100,0%	57,1%	100,0%	100,0%	100,0%	91,8%	98,0%	0,0%	0,0%
p-value^$^	<0.001	*0*.*359*	<0.001	<0.001	<0.001	<0.001	<0.001	<0.001	<0.001	*0*.*051*	*0*.*423*	*0*.*423*

The percentage of articles that adequately reported PRISMA for Abstract items per journal type, ENT journals-vs- CDSR.

^$^ Chi2 test. P-values in italic typeface highlight a difference that was *not* statistically significantly different between the two journal types.

Only ~10% of articles published in ENT journals reported the methods of their studies adequately (items 3–5, Eligibility criteria, Information sources and Risk of bias, respectively). Moreover, the strengths and limitations (item 9) of their studies are adequately discussed in the abstracts of only one quarter of the studies.

In contrast to the full text assessment, SRs published in the CDSR reported individual items of the PRISMA for Abstracts checklist inadequately more frequently. For instance, often a specific outcome was not reported in the abstract, therefore failing to adequately report Objectives (item 2) according to the PICOS structure (Patient, Intervention, Comparison, Outcome and Study design). Also the assessment of the risk of bias was not always reported in the abstracts. Although it had always been performed, we assessed whether it was reported adequately in the abstract or not. Finally, SRs and MAs published in both ENT journals and in the CDSR failed to report details on funding and registration in their abstracts (items 11 and 12).

In sum, the articles published in ENT journals reported a median of 41.7% (mean 31.7%, 30.2%-44.0%) of PRISMA for Abstracts items adequately, whereas the articles published in the CDSR reported a median of 75.0% (mean 75.2%, 73.1%-76.2%) adequately. The difference in the reporting of PRISMA for Abstracts items between the two journal types is statistically significant (*p* < 0.001).

## Discussion

To our knowledge, this study is the first to assess the quality of reporting of SRs and MAs in otorhinolaryngologic literature. We found that the current quality of reporting is suboptimal and we identified areas in which improvement is needed. To help authors improve their reporting, the PRISMA Statement was developed. Articles by authors who adhered to the PRISMA Statement were associated with improved quality of reporting [6–10].

We found several individual items of the PRISMA checklist that require extra attention (Fig 2) in the reporting of SRs and MAs published in ENT journals. First, an unpublished study protocol reduces reproducibility and prohibits comparison of the intended and final review procedures. Therefore, the review is prone to publication bias [14]. Recently, the PRISMA-P (protocol) initiative was launched, helping authors with developing a review protocol [15, 16]. Second, if risk of bias within and across studies is not assessed and the quality of the included studies is not critically appraised, readers are unable to value the results of the review. Finally, it is important to be transparent about the financial support that the review received, as specific types of sponsorship may be associated with positive study results [17]. The International Committee of Medical Journal Editors (ICMJE) has published a conflicts of interest form and checklist (http://www.icmje.org/conflicts-of-interest/), which may help in improving the transparency of financial support of review authors.

In our study, we identified a significant difference between the quality of reporting of SRs and MAs published in ENT journals versus those published in the CDSR: medians of 54.4% versus 100.0% of PRISMA items were reported adequately. In other research fields, similar deficits were identified previously. For example, in dentistry, orthodontics and radiology, the reporting of trial registration, funding and risk of bias within and across studies was suboptimal [7, 9, 18]. In line with our findings, the quality of reporting of SRs and MAs published in the CDSR was significantly better than that of SRs and MAs published in subject specific journals [7, 16, 19].

This study also assessed PRISMA for Abstracts items separately. The methodology and discussion section of abstracts are generally not reported adequately by SRs and MAs published in ENT journals (Fig 3). Abstracts of SRs and MAs published in ENT journals reported a median of 41.7% of PRISMA for Abstracts items adequately versus 75.0% for SRs and MAs published in CDSR. An extensive recent study by Hopewell et al. found many inadequately reported items (participants, harms, strengths and limitations and funding source) in conference abstracts [20].

Compliance with reporting guidelines (either QUORUM or PRISMA) is associated with improved reporting [5–10, 19]. Therefore, we think it is important that journals editors endorse reporting guidelines to help authors improve reporting. The CDSR already refers to the PRISMA Statement in their instructions to authors. However, none of the top 5 ENT journals endorse the PRISMA Statement (www.prisma-statement.com/endorsers.htm), nor do they refer to the Statement in the instructions to authors section on their websites.

A strength of our study is that all items were scored separately by two authors, and an independent otorhinolaryngologist was consulted if no consensus was reached. Moreover, the two authors independently selected the studies to be included. Furthermore, we included SRs and MAs from 2012 and 2013, resulting in a sufficient number of articles for our analyses. Lastly, we transparently provided all details of the search (Fig 1 and S1 File) and the scoring of items (S2 and S3 Files).

Our study also has limitations. One may wonder if the comparison of SRs and MAs published in ENT journals to SRs and MAs published in the CDSR is valid. We do not advocate that all SRs and MAs in otorhinolaryngology should be Cochrane SRs because of their superior methodological quality, because the process of writing a Cochrane SR is time-consuming and the length of Cochrane SRs may deter clinicians from using them. However, the quality of reporting of SRs and MAs published in the CDSR is superior to the quality of reporting of SRs and MAs published in ENT journals. The PRISMA checklist is a useful tool to help authors report SRs and MAs better. This analysis of the quality of reporting of SRs and MAs in ENT may serve as a benchmark for future assessments of the quality of reporting of SRs and MAs in otorhinolaryngology.

## Recommendation

Transparent reporting of what was done and found in SRs and MAs ensures that the clinician can value the results. To help authors improve the quality of reporting of SRs and MAs, the PRISMA Statement was developed [3, 4]. We advise authors to report their otorhinolaryngologic SRs and MAs according to the PRISMA Statement, as adherence to the PRISMA Statement is associated with improved quality of reporting [6–10]. We suggest to implement the PRISMA-P Statement in the development of a protocol for a SR or MA [15, 16]. Furthermore, we recommend editors of ENT journals to endorse the PRISMA Statement in the Instructions to Authors section on their websites. A next step to raise awareness of the importance of adequate reporting is an active implementation strategy of reporting guidelines. This strategy has been shown to be more effective in improving the quality of reporting, but this requires editorial effort and time [21, 22].

## Conclusion

The quality of reporting of SRs and MAs in ENT journals is suboptimal compared to the quality of reporting in CDSR. Large differences in individual items exist. As reporting according to the PRISMA Statement is associated with improved quality of reporting, authors and editors of ENT journals should adhere to the PRISMA Statement.

## Supporting Information

S1 File Complete search syntax. Date of search: 3 September 2014. * We adapted the Cochrane ENT search strategy [11] to function in the PubMed database.
(DOCX)

S2 File Explanation of criteria to score as ‘adequately reported PRISMA item’. See also Moher et al. [3] for the original PRISMA Statement and Liberati et al. [4] for the *Explanation and Elaboration* document, including guidelines for scoring. We scored all items as ‘adequately reported’ or inadequately reported’, i.e. there was no category ‘partially adequately reported’. ^$^ Since all manuscripts in the Cochrane Database of Systematic Reviews (CDSR) are systematic reviews (SRs), all titles of SRs published in the CDSR were scored as adequately reported. ^$$^ All Cochrane SRs reported that a protocol existed, as Cochrane SRs require a previously published review protocol. These are all available as the first version of the manuscript in the Cochrane Library. * Optional items, e.g. “if done”. If possible in the study and adequately reported, the item was scored as ‘adequately reported’. If possible, but not reported, the item was scored as ‘inadequately reported’. If not possible, the item was not scored as ‘inadequately reported’. ^#^ The correction for item 21 from the PRISMA website was implemented: *Present the main results of the review*. *If meta-analyses are done*, *include for each*, *confidence intervals and measures of consistency* (http://www.prisma-statement.org/statement.htm).
(DOCX)

S3 File Explanation of criteria to score as ‘adequately reported PRISMA for Abstract item’. See also Beller et al. [13] for the original PRISMA for Abstracts Statement. We scored all items as ‘adequately reported’ or inadequately reported’, i.e. there was no category ‘partially adequately reported’. ^$^ Since all manuscripts in the Cochrane Database of Systematic Reviews (CDSR) are systematic reviews (SRs), all titles of SRs published in the CDSR were scored as adequately reported.
(DOCX)

## References

[1] Oxford Centre for Evidence-based Medicine—Levels of Evidence (March 2009) Available: http://www.cebm.net/oxford-centre-evidence-based-medicine-levels-evidence-march-2009/ Accessed 18 November 2014

[2] Moher D, Cook DJ, Eastwood S, Olkin I, renne D, Stroup DE (1999) Improving the quality of reports of meta-analyses of randomised controlled trials: the QUOROM statement. Quality of Reporting of Meta-analyses. Lancet 354(9193):1896–1900. 10584742 10.1016/s0140-6736(99)04149-5

[3] Moher D, Liberati A, Tetzlaff J, Altman DG for the PRISMA Group (2009) Preferred reporting items for systematic reviews and meta-analyses: the PRISMA statement. PLoS Med 6(7):e10000097 10.1371/journal.pmed.1000097 PMC2707599 19621072

[4] Liberati A, Altman DG, Tetzlaff J, Mulrow C, Gøtzsche PC, Ioannidis JP, et al (2009) The PRISMA statement for reporting systematic reviews and meta-analyses of studies that evaluate healthcare interventions: explanation and elaboration. BMJ 21;339:b2700 10.1136/bmj.b2700 19622552 PMC2714672

[5] Delaney A, Bagshaw SM, Ferland A, Manns B, Laupland KB, Doig CJ (2005) A systematic evaluation of the quality of meta-analyses in the critical care literature. Crit Care 9(5):R575–82. 16277721 10.1186/cc3803 PMC1297628

[6] Tao KM, Li XQ, Zhou QH, Moher D, Ling CQ, Yu WF (2011) From QUOROM to PRISMA: a survey of high-impact medical journals' instructions to authors and a review of systematic reviews in anesthesia literature. PLoS One 6(11):e27611 10.1371/journal.pone.0027611 22110690 PMC3217994

[7] Fleming PS, Seehra J, Polychronopoulou A, Fedorowicz Z, Pandis N (2013) A PRISMA assessment of the reporting quality of systematic reviews in orthodontics. Angle Orthod 83(1):158–63. 10.2319/032612-251.1 22720835 PMC8805538

[8] Panic N, Leoncini E, de Belvis G, Ricciardi W, Boccia S (2013) Evaluation of the endorsement of the preferred reporting items for systematic reviews and meta-analysis (PRISMA) statement on the quality of published systematic review and meta-analyses. PLoS One 8(12):e83138 10.1371/journal.pone.0083138 24386151 PMC3873291

[9] Tunis AS, McInnes MD, Hanna R, Esmail K (2013) Association of study quality with completeness of reporting: have completeness of reporting and quality of systematic reviews and meta-analyses in major radiology journals changed since publication of the PRISMA statement? Radiology 269(2):413–26. 10.1148/radiol.13130273 23824992

[10] Stevens A, Shamseer L, Weinstein E, Yazdi F, Turner L, Thielman J, et al (2014) Relation of completeness of reporting of health research to journals' endorsement of reporting guidelines: systematic review. BMJ 348:g3804 10.1136/bmj.g3804 24965222 PMC4070413

[11] The editorial team, Cochrane Ear Nose and Throat Disorders Group. About the Cochrane Collaboration (Cochrane Reviews Group (CRGs)), 2012 issue 7, art. no.: ENT. CENTRAL search strategy. Available: http://onlinelibrary.wiley.com/o/cochrane/clabout/articles/ENT/sect0-meta.html. Accessed 3 September 2014.

[12] National Institutes of Health, US National Library of Medicine. Search Strategy Used to Create the Systematic Reviews Subset on PubMed, Available: http://www.nlm.nih.gov/bsd/pubmed_subsets/sysreviews_strategy.html, Accessed 3 September 2014.

[13] Beller EM, Glasziou PP, Altman DG, Hopewell S, Bastian H, Chalmers I, et al for the PRISMA for Abstracts Group (2013) PRISMA for Abstracts: reporting systematic reviews in journal and conference abstracts. PLoS Med 10(4):e1001419 10.1371/journal.pmed.1001419 23585737 PMC3621753

[14] Silagy CA, Middleton P, Hopewell S (2002) Publishing protocols of systematic reviews: comparing what was done to what was planned. JAMA 287(21):2831–4. 12038926 10.1001/jama.287.21.2831

[15] Moher D, Shamseer L, Clarke M, Ghersi D, Liberati A, Petticrew M, et al for the PRISMA-P Group (2015) Preferred reporting items for systematic review and meta-analysis protocols (PRISMA-P) 2015 statement. Syst Rev 1;4:1 10.1186/2046-4053-4-1 25554246 PMC4320440

[16] Shamseer L, Moher D, Clarke M, Ghersi D, Liberati A, Petticrew M, et al for the PRISMA-P Group (2015) Preferred reporting items for systematic review and meta-analysis protocols (PRISMA-P) 2015: elaboration and explanation. BMJ 2;349:g7647 10.1136/bmj.g7647 25555855

[17] Sun GH, Houlton JJ, MacEachern MP, Bradford CR, Hayward RA (2013) Influence of study sponsorship on head and neck cancer randomized trial results. Head Neck 35(10):1515–20. 10.1002/hed.23151 22987508

[18] Seehra J, Fleming PS, Polychronopoulou A, Pandis N (2013) Reporting completeness of abstracts of systematic reviews published in leading dental specialty journals. Eur J Oral Sci 121(2):57–62. 10.1111/eos.12027 23489893

[19] Moher D, Tetzlaff J, Tricco AC, Sampson M, Altman DG (2007) Epidemiology and reporting characteristics of systematic reviews. PLoS Med 4(3):e78 10.1371/journal.pmed.0040078 17388659 PMC1831728

[20] Hopewell S, Boutron I, Altman DG, Ravaud P (2015) Deficiencies in the publication and reporting of the results of systematic reviews presented at scientific medical conferences. J Clin Epidemiol. 10.1016/j.jclinepi.2015.03.006 25890806

[21] Pandis N, Shamseer L, Kokich VG, Fleming PS, Moher D (2014) Active implementation strategy of CONSORT adherence by a dental specialty journal improved randomized clinical trial reporting. J Clin Epidemiol 67(9):1044–8. 10.1016/j.jclinepi.2014.04.001 24837296

[22] Hopewell S, Ravaud P, Baron G, Boutron I (2012) Effect of editors' implementation of CONSORT guidelines on the reporting of abstracts in high impact medical journals: interrupted time series analysis. BMJ 344:e4178 10.1136/bmj.e4178 22730543 PMC3382226

