# Supplementary material for: Reporting Quality of Systematic Reviews and Meta-Analyses of Otorhinolaryngologic Articles Based on the PRISMA Statement
Source: PLoS One. 2015 Aug 28;10(8):e0136540. doi: 10.1371/journal.pone.0136540 (PMC4552785; doi:10.1371/journal.pone.0136540)
Supplement: S1 File — Date of search: 3 September 2014. * We adapted the Cochrane ENT search strategy [11] to function in the PubMed database. (DOCX) [file pone.0136540.s001.docx]

**Supporting Information 1**

| **Search** | **Syntax** | **Result** |
| --- | --- | --- |
| #1 | *Search syntax for adapted* Cochrane ENT search strategy [9]*  (((((Otorhinolaryngologic diseases[MeSH Terms]) OR (Otorhinolaryngologic neoplasms[MeSH Terms]) OR (Otorhinolaryngologic surgical procedures[MeSH Terms]) OR (((mastoid*[ti] OR ossic*[ti] OR stape*[ti] OR cochlea*[ti] OR auric*[ti] OR labyrinth*[ti] OR auditory[ti] OR hearing[ti] OR ((auditory[ti] OR acoustic[ti]) AND nerve[ti]) OR vestibul*[ti] OR eardrum*[ti] OR tympan*[ti] OR "eustachian tube"[ti] OR ramsay[ti] OR (towne*[ti] AND brock[ti]) OR branchio*[ti] OR "cochleo vestibular"[ti] OR cochleovestibular[ti] OR pinna*[ti] OR acoustic[ti] OR maxillary[ti] OR jaw[ti] OR mandibular[ti] OR mandible[ti]) OR (neck[ti] OR nose[ti] OR nasal[ti] OR endonasal[ti] OR paranasal[ti] OR olfact*[ti] OR turbinat*[ti] OR choana*[ti] OR antrochoana*[ti] OR adenoid*[ti] OR postcricoid*[ti] OR intranasal[ti] OR sinus*[ti] OR smell*[ti] OR cilia*[ti] OR septal[ti] OR saliva*[ti] OR parotid[ti] OR sublingual[ti] OR submandibular[ti] OR submaxillary[ti] OR sicca[ti] OR stensen*[ti] OR (wharton*[ti] AND (gland* OR duct*))) OR (pharyn*[ti] OR nasopharyn*[ti] OR oropharyn*[ti] OR hypopharyn*[ti] OR retropharyn*[ti] OR rhinopharyn*[ti] OR parapharyn*[ti] OR palatopharyn*[ti] OR velopharyn*[ti] OR palatal[ti] OR palatine[ti] OR velopalatine[ti] OR throat[ti] OR tonsil*[ti] OR retrotonsil*[ti] OR peritonsillar[ti] OR laryn*[ti] OR voice[ti] OR "vocal cord"[ti] OR “vocal cords”[ti] OR "vocal fold"[ti] OR “vocal folds”[ti] OR glottis[ti] OR epiglot*[ti] OR supraglot*[ti] OR epilaryn*[ti] OR lemierre[ti])) AND (((disease*[ti] OR disorder*[ti]) OR (infect*[ti] OR inflamm*[ti] OR pain*[ti] OR surg*[ti] OR neoplasm*[ti] OR cancer*[ti] OR tumour*[ti] OR tumor*[ti] OR carcinom*[ti] OR treatment*[ti] OR therap*[ti] OR malignan*[ti])))))) OR (((oticus[ti] OR otoliq*[ti] OR ototox*[ti] OR otolog*[ti] OR otoplasty[ti] OR pinnaplasty[ti] OR ossiculoplasty[ti] OR staped*[ti] OR "ear wax"[ti] OR earwax[ti] OR cerumen[ti] OR "ear effusion"[ti] OR “ear effusions”[ti] OR "glue ear"[ti] OR otosclerosis[ti] OR otospongiosis[ti] OR paracusis[ti] OR presbyacusis[ti] OR "auditory nerve"[ti] OR “auditory nerves”[ti] OR deaf[ti] OR deafness[ti] OR hyperacusis[ti] OR hypoacusis[ti] OR "hearing impaired"[ti] OR "hearing loss"[ti] OR "loudness recruitment"[ti] OR "loudness perception"[ti] OR "auditory inattention"[ti]) OR (alport[ti] OR aldrich[ti] OR brock[ti] OR gradenigo*[ti] OR "herpes zoster oticus"[ti] OR cephalicus[ti] OR "perilymph fistula"[ti] OR "perilymph fistulas"[ti] OR "cochlear hydrops"[ti] OR "endolymphatichydrops"[ti] OR (hydrops[ti] AND labyrinth*[ti]) OR ((motion[ti] OR air[ti] OR car[ti] OR travel[ti] OR sea[ti]) AND (sick*[ti])) OR seasick*[ti] OR carsick*[ti] OR dizziness[ti]) OR (earplug*[ti] OR (ear[ti] AND (plug*[ti] OR mold*[ti] OR mould*[ti])) OR (hearing[ti] AND (aid*[ti] OR device*[ti])) OR ((hearing[ti] OR vestibular[ti]) AND rehabilitation[ti]) OR "endolymphatic shunt"[ti] OR "labyrinth fenestration" OR “ear inflation”[ti] OR autoinflation[ti] OR ventilation[ti] OR (tympanostomy[ti] AND tube*[ti]) OR grommet*[ti] OR myringostomy[ti] OR myringotomy[ti] OR myringoplasty[ti] OR epley[ti]) OR (((hearing[ti] OR vestibular[ti] OR caloric[ti] OR barany*[ti] OR “acoustic impedence”[ti]) AND test*[ti]) OR "dichotic listening"[ti] OR audiometr*[ti] OR audiography[ti] OR audiology[ti] OR otoscopy[ti] OR tonotopy[ti] OR electronystagmography[ti] OR cochleostomy[ti] OR mastoidectomy[ti] OR ((auditory[ti] OR ear[ti] OR cochlea*[ti]) AND (implant[ti] OR implants[ti] OR implantation*[ti]))) OR ("nasal provocation"[ti] OR "nose provocation"[ti] OR epistax*[ti] OR nosebleed*[ti] OR ((nose[ti] OR nasal[ti) AND bleed*[ti]) OR (nasal[ti] AND liquorr*[ti]) OR anosmi*[ti] OR cacosmi*[ti] OR dysosmi*[ti] OR hyposmi*[ti] OR paraosmi*[ti] OR (anteverted[ti] AND (nares[ti] OR nostril*[ti])) OR antritis[ti] OR rhinitis[ti] OR pollinosis[ti] OR pollenosis[ti] OR hayfever[ti] OR “hay fever”[ti] OR rhinolog*[ti] OR rhinoman*[ti] OR rhinosinusitis[ti] OR rhinoconjunctivit*[ti]) OR (rhinolaryn*[ti] OR rhinopharyn*[ti] OR rhinoscler*[ti] OR rhinoscop*[ti] OR rhinosept*[ti] OR adenoiditis[ti] OR hypernasality[ti] OR hyponasality[ti] OR ((cleft[ti] AND lip[ti]) AND (nose[ti] OR nasal[ti] OR nostril[ti])) OR sinusitis[ti] OR parasinusitis[ti] OR snoring[ti] OR snore[ti] OR sneez*[ti] OR esthesioneuroblastoma[ti] OR "nasal glioma"[ti] OR "nasal heterotopia"[ti] OR (choan*[ti] AND (atresia[ti] OR stenosis[ti]))) OR (((nose[ti] OR nasal[ti] OR sinus[ti]) AND (scleroma[ti] OR mucocele[ti])) OR dacryocystorhinostomy[ti] OR septoplasty[ti] OR septorhinoplasty[ti] OR adenoidectomy[ti] OR ((nasal[ti] OR nose[ti]) AND (pack*[ti] OR tampon*[ti] OR decongest*[ti] OR drops[ti]))) OR (((pharyn*[ti] OR laryn*[ti] OR oesophageal[ti] OR esophageal[ti]) AND reflux[ti]) OR pharyngectomy[ti] OR pharyngostomy[ti] OR tonsillectomy[ti] OR tonsillotomy[ti] OR pharyngoplasty[ti] OR velopharyngoplasty[ti] OR nasopharyn*[ti] OR pharyngitis[ti] OR quinsy[ti] OR "peritonsillar abscess"[ti] OR tonsillitis[ti] OR tonsilitis[ti] OR "globuspharyngeus"[ti] OR "sore throat"[ti] OR epipharyngitis[ti] OR laryngitis[ti] OR uvulopalatopharyngoplasty[ti] OR uvulopharyngopalatoplasty[ti] OR uppp[ti]) OR (((pharyn*[ti] OR zenker*[ti] OR esophagopharyn*[ti]) AND (divert* OR pouch*[ti])) OR laryngismus[ti] OR laryngomalacia[ti] OR laryngitis[ti] OR (laryn*[ti] AND (malacia[ti] OR web[ti] OR dystonia[ti] OR speech[ti] OR granuloma[ti])) OR dysphoni*[ti] OR aphoni*[ti] OR hoarse*[ti] OR laryngocele[ti] OR laryngostenosis[ti] OR laryngotrach*[ti] OR laryngospasm*[ti]) OR (”upper respiratory tract infection"[ti] OR (upper[ti] AND (airway*[ti] OR respiratory[ti]) AND infection*[ti]) OR (urt[ti] AND (inflamm*[ti] OR infect*[ti])) OR urti[ti] OR parotitis[ti] OR parotiditis[ti] OR sialadenitis[ti] OR sialodenitis[ti] OR sialitis[ti] OR asialia[ti] OR hyposalivation[ti] OR hypersalivation[ti] OR hyposialorrh*[ti] OR hypersialorrh*[ti] OR ptyalism[ti] OR sialosis[ti] OR sialometaplasia[ti] OR branchiom*[ti] OR "glomuscaroticum"[ti] OR "carotid paraganglioma"[ti]))))) | 473,261 |
| #2 | *Search Strategy Used to Create the Systematic Reviews Subset on PubMed [10]*  ((systematic review [ti] OR meta-analysis [pt] OR meta-analysis [ti] OR systematic literature review [ti] OR (systematic review [tiab] AND review [pt]) OR consensus development conference [pt] OR practice guideline [pt] OR cochrane database syst rev [ta] OR acp journal club [ta] OR health technol assess [ta] OR evid rep technol assess summ [ta] OR drug class reviews [ti]) OR (clinical guideline [tw] AND management [tw])OR ((evidence based[ti] OR evidence-based medicine [mh] OR best practice* [ti] OR evidence synthesis [tiab]) AND (review [pt] OR diseases category[mh] OR behavior and behavior mechanisms [mh] OR therapeutics [mh] OR evaluation studies[pt] OR validation studies[pt] OR guideline [pt] OR pmcbook)) OR ((systematic [tw] OR systematically [tw] OR critical [tiab] OR (study selection [tw]) OR (predetermined [tw] OR inclusion [tw] AND criteri* [tw]) OR exclusion criteri* [tw] OR main outcome measures [tw] OR standard of care [tw] OR standards of care [tw]) AND (survey [tiab] OR surveys [tiab] OR overview* [tw] OR review [tiab] OR reviews [tiab] OR search* [tw] OR handsearch [tw] OR analysis [ti] OR critique [tiab] OR appraisal [tw] OR (reduction [tw]AND (risk [mh] OR risk [tw]) AND (death OR recurrence))) AND (literature [tiab] OR articles [tiab] OR publications [tiab] OR publication [tiab] OR bibliography [tiab] OR bibliographies [tiab] OR published [tiab] OR unpublished [tw] OR citation [tw] OR citations [tw] OR database [tiab] OR internet [tiab] OR textbooks [tiab] OR references [tw] OR scales [tw] OR papers [tw] OR datasets [tw] OR trials [tiab] OR meta-analy* [tw] OR (clinical [tiab] AND studies [tiab]) OR treatment outcome [mh] OR treatment outcome [tw] OR pmcbook)) NOT (letter [pt] OR newspaper article [pt] OR comment [pt])) | 222,423 |
| #3 | *Search syntax for date restriction*  (“2012”[Date – Publication] : “2013”[Date – Publication]) | 2,060,545 |
| #4 | *Search syntax for Cochrane Database of Systematic Reviews*  “Cochrane Database Syst Rev”[jour] | 10,282 |
| #5 | *Search syntax for 2013 top 5 ENT journals*  (“Ear Hear”[jour] OR “J Assoc Res Otolaryngol”[jour] OR “Head Neck”[jour] OR “Hear Res”[jour] OR “Rhinology”[Jour]) | 14,883 |
| #6 | *Search syntax to exclude publication types*  (letter[pt] OR comment[pt] OR editorial[pt] OR news[pt]) | 1,493,749 |
| #7 | *Search to retrieve SRs and MAs from Cochrane Database of Systematic Reviews*  (#1 AND #2 AND #3 AND #4) NOT #6 | 91 |
| #8 | *Search to retrieve SRs and MAs from ENT journals*  (#1 AND #2 AND #3 AND #5) NOT #6 | 36 |
