# Supplementary material for: Reporting Quality of Systematic Reviews and Meta-Analyses of Otorhinolaryngologic Articles Based on the PRISMA Statement
Source: PLoS One. 2015 Aug 28;10(8):e0136540. doi: 10.1371/journal.pone.0136540 (PMC4552785; doi:10.1371/journal.pone.0136540)
Supplement: S2 File — See also Moher et al. [3] for the original PRISMA Statement and Liberati et al. [4] for the Explanation and Elaboration document, including guidelines for scoring. We scored all items as ‘adequately reported’ or inadequately reported’, i.e. there was no category ‘partially adequately reported’. $ Since all manuscripts in the Cochrane Database of Systematic Reviews (CDSR) are systematic reviews (SRs), all titles of SRs published in the CDSR were scored as adequately reported. $$ All Cochrane SRs reported that a protocol existed, as Cochrane SRs require a previously published review protocol. These are all available as the first version of the manuscript in the Cochrane Library. * Optional items, e.g. “if done”. If possible in the study and adequately reported, the item was scored as ‘adequately reported’. If possible, but not reported, the item was scored as ‘inadequately reported’. If not possible, the item was not scored as ‘inadequately reported’. # The correction for item 21 from the PRISMA website was implemented: Present the main results of the review. If meta-analyses are done, include for each, confidence intervals and measures of consistency (http://www.prisma-statement.org/statement.htm). (DOCX) [file pone.0136540.s002.docx]

**Supporting Information 2**

| **Item** | | **Criteria to score as adequately reported** |
| --- | --- | --- |
| *Title* | | |
| 1 | Title | The title contained *systematic review* and/or *meta-analysis*.^$^ |
| *Abstract* | | |
| 2 | Abstract | See *Supporting Information 3*. |
| *Introduction* | | |
| 3 | Rationale | The rationale for the review was described in the context of what was already known. |
| 4 | Objectives | The objective of the study with reference to Patients, Interventions, Comparisons, Outcomes and Study design (PICOS) was reported. |
| *Methods* | | |
| 5 | Protocol and registration | It was described if a research protocol was available and where it could be accessed and, if available, the registration number was provided.^$$^ |
| 6 | Eligibility criteria | Study characteristics (PICOS) and report characteristics used as criteria for inclusion were specified. |
| 7 | Information sources | All information sources and date of last search were described. |
| 8 | Search | Search syntax for at least one database was presented, either in the full text or as an appendix. |
| 9 | Study selection | The process for selecting studies (screening, inclusion, critical appraisal, data analysis) was stated. |
| 10 | Data collection process | The methods for data extraction from included studies was reported. |
| 11 | Data items | The variables that were to be extracted were noted. |
| 12 | Risk of bias in individual studies | The methods to assess risk of bias of individual studies were reported. |
| 13 | Summary measures | Principal summary measures were described. |
| 14* | Synthesis of results | The methods of combining results were reported (in case of a meta-analysis) |
| 15 | Risk of bias across studies | The methods to assess risk of bias across studies (e.g. publication bias) was described. |
| 16* | Additional analyses | The methods for additional analyses were reported (in case of meta-analysis). |
| *Results* | | |
| 17 | Study selection | Numbers of studies screened, assessed and included were provided with reasons for exclusions. Ideally, a flow diagram was present, but this was not obligatory. |
| 18 | Study characteristics | Study characteristics (PICOS, number of patients, follow up time) that were extracted were described for each study. |
| 19 | Risk of bias within studies | The results of the assessment of the risk of bias (item 12) for each study were reported. |
| 20 | Results of individual studies | Summary data for each intervention group on all assessed outcomes for each study were presented. A forest plot was not obligatory. |
| 21 * ^#^ | Synthesis of results | The main results of the review are reported. In case of meta-analyses, confidence intervals and measures of consistency were described. |
| 22 | Risk of bias across studies | The results of the assessment of risk of bias across studies (item 15) are presented. |
| 23 * | Additional analyses | The results for additional analyses (item 16) were described. |
| *Discussion* | | |
| 24 | Summary of evidence | A summary of evidence was provided. |
| 25 | Limitations | Limitations at the level of included studies were discussed, as well as limitations at review level. |
| 26 | Conclusions | A general interpretation of the results was provided and directions for future research were stated. |
| *Funding* | | |
| 27 | Funding | Sources of funding or other support and their roles in the review process were described. |
