# Supplementary material for: Reporting Quality of Systematic Reviews and Meta-Analyses of Otorhinolaryngologic Articles Based on the PRISMA Statement
Source: PLoS One. 2015 Aug 28;10(8):e0136540. doi: 10.1371/journal.pone.0136540 (PMC4552785; doi:10.1371/journal.pone.0136540)
Supplement: S3 File — See also Beller et al. [13] for the original PRISMA for Abstracts Statement. We scored all items as ‘adequately reported’ or inadequately reported’, i.e. there was no category ‘partially adequately reported’. $ Since all manuscripts in the Cochrane Database of Systematic Reviews (CDSR) are systematic reviews (SRs), all titles of SRs published in the CDSR were scored as adequately reported. (DOCX) [file pone.0136540.s003.docx]

**Supporting Information 3**

| **Item** | | **Criteria to score as adequately reported** |
| --- | --- | --- |
| *Title* | | |
| 1 | Title | The title contained *systematic review* and/or *meta-analysis*.^$^ |
| *Background* | | |
| 2 | Objectives | The objective of the study with reference to Patients, Interventions, Comparisons, Outcomes and Study design (PICOS) was reported. |
| *Methods* | | |
| 3 | Eligibility criteria | Study characteristics (PICOS) and report characteristics used as criteria for inclusion were specified. |
| 4 | Information sources | Information sources and date of last search were described. |
| 5 | Risk of bias | Methods to assess risk of bias were reported. |
| *Results* | | |
| 6 | Included studies | Number and type of included studies and participants were reported. |
| 7 | Synthesis of results | Results for main outcomes were presented. |
| 8 | Description of the effect | Direction and size of the effect was reported. |
| *Discussion* | | |
| 9 | Strengths and Limitations of the evidence | Strengths and limitations of the evidence were discussed. |
| 10 | Interpretation | Results and implications were interpreted. |
| *Other* | | |
| 11 | Funding | Source of funding for the review was stated. |
| 12 | Registration | Registry name and registration number was provided. |
